# Frailty, transition in frailty status and all-cause mortality in older adults of a Taichung community-based population

**DOI:** 10.1186/s12877-019-1039-9

**Published:** 2019-01-28

**Authors:** Mu-Cyun Wang, Tsai-Chung Li, Chia-Ing Li, Chiu-Shong Liu, Wen-Yuan Lin, Chih-Hsueh Lin, Chuan-Wei Yang, Shing-Yu Yang, Cheng-Chieh Lin

**Affiliations:** 1 Department of Family Medicine, China Medical University Hospital, Taichung, Taiwan; 2 School of Medicine, College of Medicine, China Medical University, Taichung, Taiwan; 3 Department of Public Health, College of Public Health, China Medical University, Taichung, Taiwan; 4 Department of Healthcare Administration, College of Medical and Health Science, Asia University, Taichung, Taiwan; 5 Department of Medical Research, China Medical University Hospital, Taichung, Taiwan; 6 China Medical University, 91 Hsueh-Shih Road, Taichung, 40421 Taiwan

**Keywords:** Frailty, Transition in frailty status, Fried’s phenotype, Mortality

## Abstract

**Background:**

Previous studies have reported the associations of frailty phenotype or its components with mortality. However, studies that explored the effects of transition in frailty status on mortality were far less in Asian or Chinese. The aim of this study was to evaluate baseline frailty status and one-year change of frailty status in relation to all-cause mortality in Taiwanese community-dwelling older adults who participated in the Taichung Community Health Study for Elders.

**Methods:**

We conducted a community-based prospective cohort study. A total of 921 community-dwelling elderly men and women aged 65–99 years in Taichung City were enrolled in 2009–2010 and were followed up through 2016. We adopted the definition of frailty proposed by Fried et al., including five components: shrinking, weakness, poor endurance and energy, slowness, and low physical activity. Cox proportional hazards models were used to determine adjusted hazard ratios (HRs) of mortality with 95% confidence intervals (CIs) for frailty at baseline and one-year change in frailty status.

**Results:**

There were 160 deaths during the follow-up period. The mortality rates in groups of robust and frail were 20.26 and 84.66 per 1000 person-years respectively. After multivariate adjustment, the HR (CIs) for baseline frailty was 2.67 (1.73–4.12). Poor endurance and energy [1.88 (1.03–3.42)], slowness [2.60 (1.76–3.83)] and weakness [1.65 (1.16–2.33)] were found to be predictors of mortality. Increased risks in mortality for subgroups of robust-to-frail [2.76 (1.22–6.27)], frail-to-robust [3.87 (1.63, 9.19)], and frail-to-frail [4.08 (1.92–8.66)] over one-year period were observed compared with those remaining robust.

**Conclusion:**

Baseline frailty status and one-year change in frailty status are associated with 6-year all-cause mortality among Taiwanese elderly adults. Frailty may be useful for identifying older adults at high risks for mortality prevention.

## Background

Taiwan has the fastest ageing population in the world with a time span of 25 years from an aging society to an aged society compared with those of 60–100 years in Europe and the USA [1]. In response to the care needs due to the fast ageing population, identifying older adults at high risks of adverse outcomes and targeting prevention interventions toward them have become very important issues.

Frailty, a dynamic and complex geriatric state, has been used to describe very old individuals in a state of increased vulnerability to stressors resulting from multisystem decline in reserve and function including impaired strength, fatigue, weight loss, slowed motor processing and performance, social withdrawal, decreased balance, impaired cognition, and diminished physical functioning [2]. Although there have been several instrument to operationalize frailty, most were built on two common models. Fried et al. proposed frailty phenotype to define frailty as the presence of three or more of the following components: weakness, weight loss, slow walking speed, fatigue, and low levels of activity [3]. Rockwood et al. developed so-called frailty index based on deficit accumulation [4]. Frailty adds a clinical value in prognosis and decision-making because frail persons are vulnerable to many adverse outcomes such as impairment of function, infection, falls, institutionalization, and death [5], and frail older persons have adverse pathophysiologic or functional changes not captured fully by comorbidity and disability definitions.

Frailty has independent predictive value for mortality, even after adjusting for disease diagnoses and other factors [3]. We found several studies reporting frailty or its components in association with mortality in Western populations [3, 6–14], Asian [15–17] or Chinese [18, 19]. Many studies adopted Fried’s phenotype as the operational definition of frailty for its precision and practicability. There were only two studies that examined the association between frailty index and mortality in middle-aged Chinese in Taiwan [18] and in Hong Kong [19]. In addition, few studies explored the effects of frailty components on mortality [10, 11]. To consider frailty in a dynamic process, the association between transition in frailty index and mortality had been reported [20, 21]. However, only one research in Mexican Americans reported transition in frailty status using modified Fried’s phenotype [22]. The aims of this study were to explore baseline frailty status and transition in frailty status during the first year in relation to all-cause mortality in older adults who participated in the Taichung Community H ealth Study for Elders (TCHS-E) during a 6-year follow-up period.

## Methods

### Study design and participants

A community-based prospective cohort study was conducted among all participants of TCHS-E [23]. The target population of TCHS-E consisted of all residents aged 65 and above in eight administrative neighborhoods of North District of Taichung City, Taiwan, in June 2009. All eligible individuals were invited to participate in the study. Participants provided information through face-to-face interviews at physical check-up visits in the years 2009 and 2010. The age and gender distributions of these eight administrative neighborhoods were similar to those of Taichung City and Taiwan populations at the time of survey. A total of 3997 elderly residents were in the study population in the year 2009. A total of 1247 individuals were found to be not eligible during household visits and were excluded from the study sample. The reasons for exclusion included moving out of the area (*n*  =  949), institutionalization (*n*  =  52), deaths (*n*  =  122), and errors of the registry (*n*  =  124). Among 2750 eligible participants, 1347 agreed to participate with an overall response rate of 49.0% at the baseline. Among these, 1078 participants remained to be accessed in the year 2010. The reasons for attrition included deaths (*n* = 21), institutionalization (*n* = 2), and refusal of interview (*n* = 246). The measurements of frailty status and number of components for frailty in 2009 were used as baseline values, and the measurements in 2010 were used to derive changes in frailty status. Excluding those with missing data, 921 were left for the analysis of baseline frailty and 597 remained for the analysis of frailty change. All participants were followed up until death or December 31, 2016. TCHS-E was sponsored by the National Health Research Institute, and was approved by the Human Research Committee (HRC) (DMR 97-IRB-055). Written informed consent was obtained from each participant for the first and second waves of data collection. The present study was also approved by HRC (CMUH105-REC1–026), the informed consent was not required because the study was a secondary data analysis.

### Measurements

#### Socio-demographic factors, lifestyle behaviors, and chronic diseases

Standardized questionnaires had been used to collect socio-demographic factors, lifestyle behaviors, and chronic diseases in 2009. Data on socio-demographic characteristics included age, sex, educational attainment, physical activity, smoking, alcohol drinking, self-reported physician-diagnosed diseases, fall history, sleep impairment, and medication history in the past year. Participants who exercised for at least 30 min three times per week during the preceding 6 months were classified as having regular exercise for recreational physical activity. We validated regular exercise status with a single item of the habit of leisure time activity, resulting in a concordance rate of 91.0%. This high concordance rate indicated that the regular exercise status defined in this study had concurrent validity. Smoking was categorized as never, current, and former.

#### Anthropometric measurements

With complete physical examinations, anthropometric measurements and blood samples for participants in the TCHS-E were collected in 2009. Weight and height were measured with an autoanthropometer (super-view, HW-666), with participants shoeless and wearing light clothing. Body mass index (BMI) was calculated by weight (in kilograms) divided by square of height (in meters). BMI was further categorized into four classes based on the World Health Organization definition: 1) underweight: BMI < 18.5 kg/m^2^; 2) normal weight: 18.5 ≤ BMI < 25 kg/m^2^; 3) overweight: 25 ≤ BMI < 30 kg/m^2^; 4) obesity: BMI ≥30 kg/m^2^.

#### Frailty measures and death ascertainment

Frailty was defined on the basis of well-established, standardized, and widely accepted phenotype described by Fried et al., which consisted of five components: shrinking, weakness, poor endurance and energy, slowness, and low physical activity level [3]. Here, four of the five frailty components were exactly the same as those proposed by Fried, except shrinking was adapted. Older adults who had unintentional weight loss of ≥3 kg in the prior year were defined as shrinking. Weakness was defined as grip strength in the lowest quintile, measured by a handheld dynamometer (TTM110D; TTM, Tokyo, Japan), based on subgroups of gender and BMI [3]. Poor endurance and energy was indicated by self-reported exhaustion, identified by two questions from the Center for Epidemiological Studies-Depression Scale [24]. Slowness was defined as the slowest quintile of the population based on time to walk 15 ft, adjusting for gender and standing height [3]. Low physical activity level was defined as the lowest quintile of physical activity for each gender, calculated by a weighted score of kilocalories expended per week based on report of each participant [25]. Participants who presented with of three or more components were considered as frail, whereas those with less than three components were considered as robust. It should be emphasized that an intermediate state, so called pre-frailty, was not considered here. Hence the robust ones consisted of more than those with none of the components. Deaths were ascertained by using computer linkage with a unique identification number to the national database from the Health and Welfare Data Science Center, Ministry of Health and Welfare. This database records all deaths of Taiwan citizens, which are coded from death certificates.

### Statistical analysis

Simple descriptive analyses such as mean, standard deviation (SD), proportion, chi-square test and t-test were employed when appropriate. Kaplan–Meier cumulative incidence plots were derived. For multivariate analysis, Cox proportional hazards models were used to calculate the hazard ratios (HRs) and their 95% confidence intervals (CIs). The key independent variables were baseline frailty status and one-year transition in frailty status. The proportionality assumption is tested by including an interaction term of each baseline variable and follow-up time and the proportionality assumption was held. The areas under the receiver operating characteristic (AUROC) curves were calculated to evaluate the relative ability of frailty indicators to correctly classify mortality status. All analyses were performed with SAS version 9.4 (SAS, Cary, NC). All *p*-values were two tailed, and a *p*-value < 0.05 was considered statistically significant.

## Results

With an average of 6.62 years of follow-up, 160 older adults died with a crude rate of 26.26 per 1000 person-years (33.10 for men, 19.11 for women). Table 1 presents the distributions of baseline factors and frailty according to mortality status. Compared with older adults who died, those who survived had lower prevalence of frailty and higher prevalence of physical activity (all *p* < 0.05).

**Table 1:** The comparisons of baseline socio-demographic factors, lifestyle behaviors, disease history, cognitive function and frailty status according to mortality status in older adults

Variables	Mortality status N (%)		*p* value
	Alive (*N* = 761)	Dead (*N* = 160)	
Socio-demographic factors			
Men	375 (49.28)	103 (64.38)	< 0.001
Age (years)	73.15 ± 5.79	78.11 ± 7.86	< 0.001
65–74	478 (62.81)	62 (38.75)	
75–84	248 (32.59)	64 (40.00)	
> 85	35 (4.60)	34 (21.25)	
Education			0.005
≤ 6 years	293 (38.50)	72 (45.00)	
7–12 years	272 (35.74)	36 (22.50)	
≥ 13 years	196 (25.76)	52 (32.50)	
Married	552 (72.54)	107 (66.88)	0.18
Body mass index (kg/m^2^)	24.48 ± 3.35	23.74 ± 4.01	0.03
< 18.5	24 (3.15)	13 (8.13)	
18.5–25	436 (57.29)	93 (58.13)	
25–30	259 (34.03)	47 (29.38)	
≥ 30	42 (5.52)	7 (4.38)	
Lifestyle behaviors			
Smoking	70 (9.20)	14 (8.75)	0.98
Alcohol drinking	96 (12.61)	21 (13.13)	0.96
Physical activity	564 (74.11)	102 (63.75)	0.01
Disease history			
Hypertension	378 (49.67)	93 (58.13)	0.06
Diabetes Mellitus	113 (14.85)	39 (24.38)	0.005
Heart disease	206 (27.07)	72 (45.00)	< 0.001
Hyperlipidemia	203 (26.68)	27 (16.88)	0.01
Gout	79 (10.38)	24 (15.00)	0.12
Hyperuricemia	74 (9.72)	21 (13.13)	0.25
Arthritis	152 (19.97)	31 (19.38)	0.95
Osteoporosis	145 (19.05)	21 (13.13)	0.10
Stroke	36 (4.73)	20 (12.50)	< 0.001
Cataract	337 (44.28)	86 (53.75)	0.04
Fall history	161 (21.16)	54 (33.75)	< 0.001
Sleep impairment	343 (45.07)	73 (45.63)	0.97
MMSE			< 0.001
< 24	83 (10.91)	39 (24.38)	
≥ 24	678 (89.09)	121 (75.63)	
Frailty status	53 (6.96)	48 (30.00)	< 0.001
Frailty components			
Shrinking	90 (11.83)	31 (19.38)	0.01
Poor endurance and energy	22 (2.89)	15 (9.38)	< 0.001
Low physical activity	125 (16.43)	42 (26.25)	0.005
Slowness	227 (29.83)	104 (65.00)	< 0.001
Weakness	194 (25.49)	80 (50.00)	< 0.001

*MMSE* Mini–Mental State Examination

**Fig. 1:**
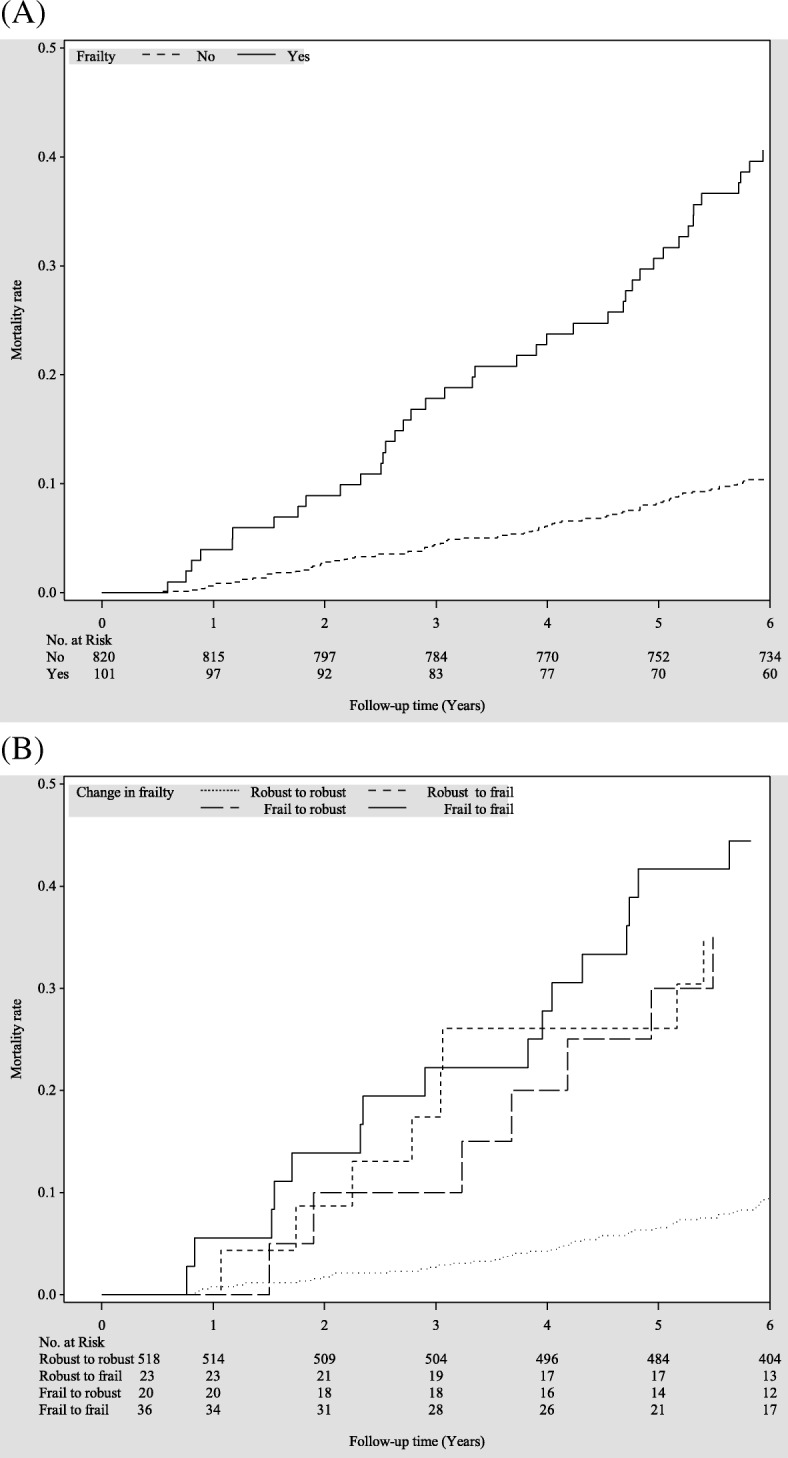
Survival curves of death for (**a**) baseline frailty status and (**b**) one-year change in frailty status

**Fig. 2:**
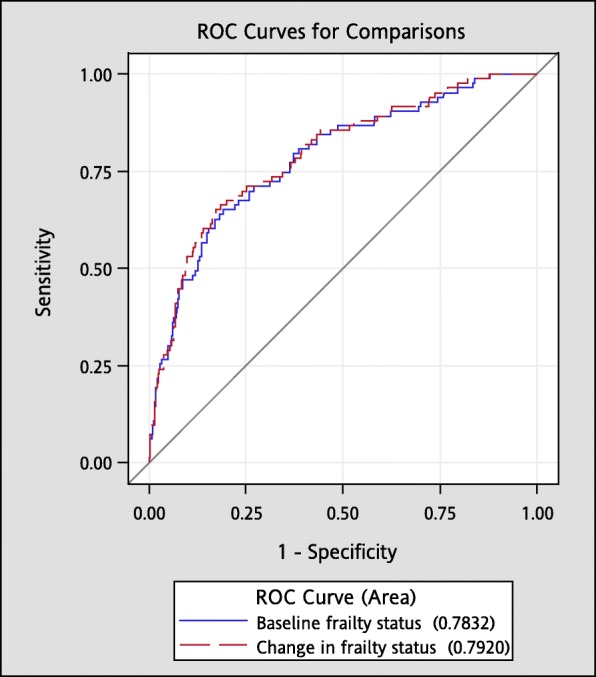
The AUROC curves for predicting all-cause mortality plotted in baseline frailty status and change in frailty status

Figure 1 presents the Kaplan–Meier survival curves for all-cause mortality within subgroups defined by baseline frailty status and one-year change in frailty status. Older adults who were frail at baseline (log-rank *p* < 0.001, Fig. 1a) and subgroups of robust-to-frail, frail-to-robust, and frail-to-frail over one-year period (log-rank *p* < 0.001, Fig. 1b) had an increased mortality risk. Table 2 presents the HRs according to baseline frailty status and transition in frailty status. After multivariate adjustment, frail older adults at baseline were associated with increased risks of all-cause mortality [HR (CI): 2.67 (1.73–4.12)]. Compared with older adults who remained robust over one year, the multivariate-adjusted HR (CI) of all-cause mortality was 2.76 (1.22–6.27), 3.87 (1.63–9.19) and 4.08 (1.92–8.66) for subgroups of robust-to-frail, frail-to-robust, and frail-to-frail. The AUROC curves demonstrated that baseline frailty status and one-year transition in frailty status had good predictive ability for all-cause mortality (both c-statistic> 0.70) (Fig. 2).

**Table 2:** The hazard ratios of all-cause mortality according to baseline frailty status and one-year transition in frailty status

Variables	N	Cases	Person-years	Incidence rate	Age and sex -adjusted	Multivariate-adjusted^1^	Multivariate-adjusted^2^
					HR (95%CI)	HR (95%CI)	HR (95%CI)
Frailty							
No	820	112	5526.80	20.26	1.00	1.00	1.00
Yes	101	48	566.95	84.66	3.24 (2.25–4.67)***	3.22 (2.13–4.87)***	2.67 (1.73–4.12)***
One-year transition in frailty status							
Robust to robust	518	51	3602.43	14.16	1.00	1.00	1.00
Robust to frail	23	8	139.23	57.46	3.78 (1.74–8.20)***	3.63 (1.65–7.97)**	2.76 (1.22–6.27)*
Frail to robust	20	7	125.07	55.97	3.43 (1.53–7.67)**	3.23 (1.38–7.53)**	3.87 (1.63–9.19)**
Frail to frail	36	17	209.31	81.22	5.13 (2.82–9.36)***	5.86 (2.93–11.70)***	4.08 (1.92–8.66)***

*:*p* < 0.05; **:*p* < 0.01; ***:*p* < 0.001

^1^Multivariate adjustment for age, sex, education, marital status, BMI, smoking, alcohol drinking, physical activity and exercising program

^2^Multivariate adjustment for age, sex, education, marital status, BMI, smoking, alcohol drinking, physical activity, exercising program, hypertension, diabetes mellitus, heart disease, hyperlipidemia, gout, hyperuricemia, arthritis, osteoporosis, stroke, cataract, fall history, sleep impairment and cognitive function

*Incidence rate* number of incident cases / person-years*1000, *HR* hazard ratio, *CI* confidence interval

**Fig. 3:**
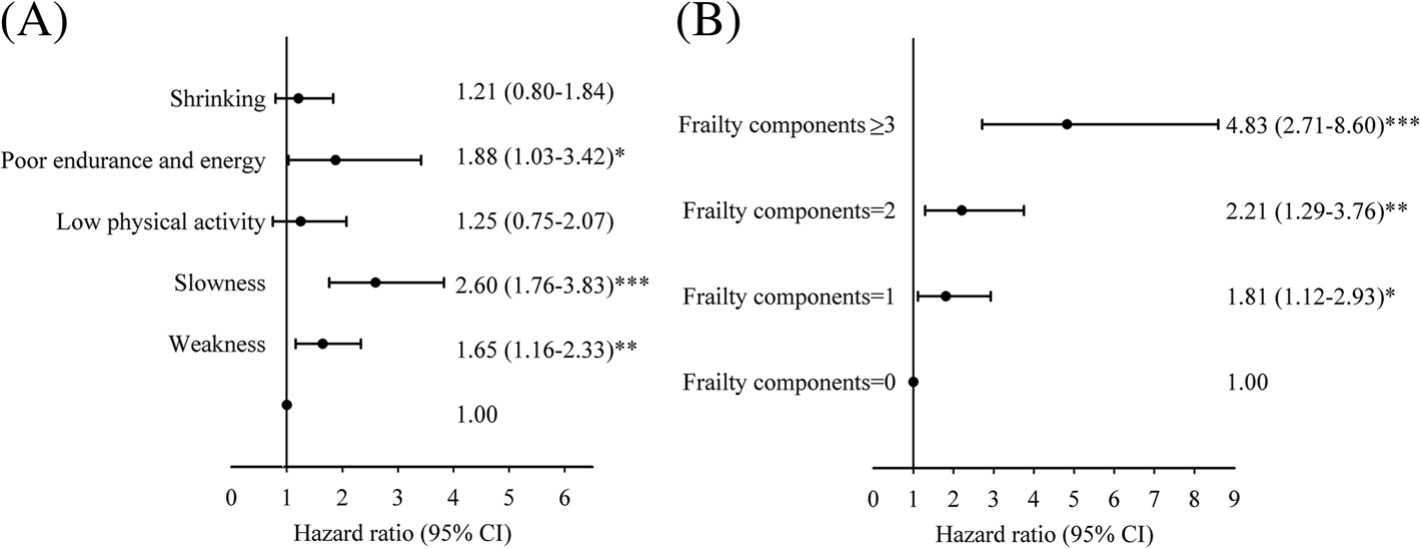
Relationship between death and (**a**) frailty components and (**b**) number of frailty components.*p:< 0.05; **:*p* < 0.01, ***:*p* < 0.001

We evaluated the effect of individual frailty components and number of frailty components and found that poor endurance and energy [HR (CI): 1.88 (1.03–3.42)], slowness [2.60 (1.76–3.83)] and weakness [1.65 (1.16–2.33)] were statistically significant predictors of all-cause mortality (Fig. 3a). Older adults with one [1.81 (1.12–2.93)], two [2.21 (1.29–3.76)] and three or more components [4.83 (2.71–8.60)] were more susceptible to all-cause mortality than those with none (Fig. 3b).

## Discussion

This study demonstrates that frailty defined by Fried et al. predicted all-cause mortality among community-dwelling older adults from a population-based cohort even after adjusting for traditional risk factors. Compared with robust older adults, frail older adults had a 2.67-fold increase in risk of death. While poor endurance and energy, slowness and weakness of these five components predicted all-cause mortality, the other components did not predict mortality. Transition in frailty status during the first year predicted all-cause mortality. Robust older adults with transition to frail status had a 2.76-fold increased mortality risk, frail older adults with transition to robust status still had a 3.87-fold increased mortality risk, and frail older adults remaining in frail status had the highest risk (a 4.08-fold increased risk). Older adults who had one or more components were associated with increased mortality, and an increasing trend was observed as the number of components increased.

Our findings regarding the predictive ability of frailty status defined by Fried et al. were consistent with prior studies. Previous meta-analyses indicated that frailty was associated with a 1.8- to 2.3-fold increased risk for mortality [11]. A 2-year prospective study based on 514 individuals aged 75 years and older in Spain reported frailty was associated with approximately fivefold increased mortality risk [26]. In a prospective cohort of 5993 community-dwelling men aged 65 years and older from six USA clinical centers, a significant association between mortality and modified Fried’s definition of frailty was observed during an average of 4.7 years of follow-up [8]. Previous longitudinal studies in Asia also have reported that frailty phenotype was associated with an odds ratio of 1.89 (1.52–2.34) for mortality in Hong Kong [19], a HR of 2.28 (1.61–3.22) for mortality in Korea [17] and a HR of 1.18 (1.06–1.33) in Latin America, India, and China [16]. In addition, Lin et al. [18] and Armstrong et al. [15] demonstrated that frailty index was associated with an increased mortality risk in older Taiwanese and participants of the Honolulu-Asia Aging Study, respectively. We report an effect size of 2.67-fold increase in mortality risk for baseline frailty, which is intermediate compared with other studies.

Conversely, some studies did not find a significant association between frailty and mortality. In a 4-year longitudinal study of 6030 French community-dwelling individuals aged 65–95 years, frailty adopting Fried’s definition was not a predictor of mortality but was associated with increased incidence of disability [6]. The possible reason that the French study could not demonstrate an association between frailty phenotype and mortality was that the sources of study participants were from three cities, which increased the heterogeneity of study participants and thus decreased the power of the study. In a prospective study of 430 community-dwelling older adults aged 70 or older with one-year follow-up, the association between three frailty tools and mortality were examined, and none of them was statistically significant [7]. This could be due to the small sample size and very short follow-up period.

Prior cohort studies have assessed the effects of frailty components on mortality and found a relative risk or HR between 1.4 and 3.6 for slowness [9, 14, 16, 27–34], between 1.4 and 3.9 for weight loss [9, 14, 16, 27, 29], between 1.5 and 3.7 for low physical activity [9, 14, 16, 27, 29], 1.8 for weak grip [14], and 1.6 for exhaustion [14]. Walking speed is considered a simple indicator of survival in older adults. Some studies have found that walking speed alone is a health indicator that can predict all-cause mortality among older persons [19, 28, 29]. Our finding also imply that walking speed as a single indicator can facilitate early diagnosis of frailty in community older adults. Stenholm et al. showed shrinkage was the latest component to develop during the natural course of frailty [35]. While weight loss often indicates acute illness, this may be the reason why shrinkage was not shown to predict mortality in our study with a relative long follow-up period of six years.

Our study’s findings indicate transition in Fried’s frailty phenotype independently predicts all-cause mortality, which is compatible with recent studies. In a cohort of 11,165 older adults from the Chinese Longitudinal Healthy Longevity Survey (CLHLS) during three years of follow-up, transition in frailty status measured by the change in score of frailty index, constructed from 44 health deficits, predicts all types of death. Older adults in “remaining frail” and “worsening” categories had an increased risk of all types of death [21]. In another study of 1171 community-dwelling Mexican Americans, Fried’s phenotype was modified with a total of four components. By their criteria, older adults with transition from pre-frail to frail, frail to pre-frail, and who remained frail over 3 years had an increased 15-year mortality risk [22]. On the contrary, a study conducting in a 3985 women cohort of the Global Longitudinal Study of Osteoporosis reported the absolute scores for frailty index measures were associated with increased risk of death in one-year follow-up, but the change in scores was not [19]. The increasing trend of mortality risk for robust-to-robust, robust-to-frail, frail-to-robust and frail-to-frail in our study implies frail baseline status alone was predictive of future mortality. The reason why those who reversed from frail to robust were still at higher risk of dying is that the robust subgroup defined here may still present with 1 or 2 frailty components. Our study was the first to use the five components of frailty phenotype proposed by Fried et al. in demonstrating that transition in frailty status was independently associated with mortality in community-based older adults.

The strengths of this study were a representative sample of community older adults, the use of standard protocols and instruments for data collection and prospective follow-up visits. To ensure the quality of data collection, a strict personnel training process and vigorous quality assurance programs were set up. On the contrary, there were some limitations. First, a small proportion of people were institutionalized, and they were excluded from the analysis. These institutionalized older adults were more likely to be frail, and possibly died. This might result in underestimating the association between frailty and mortality, however, would be a lesser threat to consistency of our study’s conclusions. Second, our sample represented a Taiwanese metropolitan elderly population, thus, our results may not be generalized to older adults residing in rural areas. Lastly, the non-participation rate was high, indicating there may exist potential selection bias. To assess this possibility, we examined the age and gender characteristics of the sample and Taichung population, and similar distributions were found. The non-differential distributions in age and sex indicate this kind of selection error might be random. The biased results in the effect may be toward the null, a lesser threat to validity.

## Conclusion

We conclude that Fried’s frailty phenotype is associated with increased risk of mortality in individuals aged 65 years and older in this community-based prospective cohort study. Our study findings indicate that older adults remaining robust over one-year period have the lowest risk of all-cause mortality. The results of this study contribute key information for the prevention of mortality in older adults. Frailty phenotype defined by Fried’s criteria may be used as a screening and monitoring instrument for primary care and active aging programs in community-dwelling older adults in Taiwan.

## Abbreviations

AUROC curves: areas under the receiver operating characteristic curves
BMI: Body mass index
CI(s): 95% confidence interval(s)
CLHLS: Chinese Longitudinal Healthy Longevity Survey
HR(s): Hazard ratio(s)
SD: Standard deviation
TCHS-E: Taichung Community Health Study for Older adults
